# Neovascular Age-Related Macular Degeneration Risk Based on *CFH, LOC387715/HTRA1, *and Smoking

**DOI:** 10.1371/journal.pmed.0040355

**Published:** 2007-12-27

**Authors:** Anne E Hughes, Nick Orr, Chris Patterson, Hossein Esfandiary, Ruth Hogg, Vivienne McConnell, Giuliana Silvestri, Usha Chakravarthy

**Affiliations:** 1 Department of Medical Genetics, Queen's University Belfast, Belfast, United Kingdom; 2 Department of Epidemiology and Public Health, Queen's University Belfast, Belfast, United Kingdom; 3 Centre for Vision Science and Vascular Biology, Queen's University Belfast, Belfast, United Kingdom; Lund University, Sweden

## Abstract

**Background:**

Age-related macular degeneration (AMD) is the major cause of blindness in the elderly. Those with the neovascular end-stage of disease have irreversible loss of central vision. AMD is a complex disorder in which genetic and environmental factors play a role. Polymorphisms in the *complement factor H* (*CFH*) gene, *LOC387715*, and the *HTRA1* promoter are strongly associated with AMD. Smoking also contributes to the etiology. We aimed to provide a model of disease risk based on these factors.

**Methods and Findings:**

We genotyped polymorphisms in *CFH* and *LOC387715/HTRA1* in a case–control study of 401 patients with neovascular AMD and 266 controls without signs of disease, and used the data to produce genetic risk scores for the European-descent population based on haplotypes at these loci and smoking history. *CFH* and *LOC387715/HTRA1* haplotypes and smoking status exerted large effects on AMD susceptibility, enabling risk scores to be generated with appropriate weighting of these three factors. Five common haplotypes of *CFH* conferred a range of odds ratios (ORs) per copy from 1 to 4.17. Most of the effect of *LOC387715/HTRA1* was mediated through one detrimental haplotype (carriage of one copy: OR 2.83; 95% confidence interval [CI] 1.91–4.20), with homozygotes being at particularly high risk (OR 32.83; 95% CI 12.53–86.07). Patients with neovascular macular degeneration had considerably higher scores than those without disease, and risk of blinding AMD rose to 15.5% in the tenth of the population with highest predicted risk.

**Conclusions:**

An individual's risk of developing AMD in old age can be predicted by combining haplotype data with smoking status. Until there is effective treatment for AMD, encouragement to avoid smoking in those at high genetic risk may be the best option. We estimate that total absence of smoking would have reduced the prevalence of severe AMD by 33%. Unless smoking habits change or preventative treatment becomes available, the prevalence of AMD will rise as a consequence of the increasing longevity of the population.

## Abstract 2

Anne Hughes and colleagues show that an individual's risk of developing age-related macular degeneration in old age can be predicted by combining haplotype data with smoking status.

## Editors' Summary

**Background.:**

Age-related macular degeneration (AMD) is the leading cause of vision loss in the elderly. The macula is the central region of the retina, the tissue at the back of the eye that converts light into electrical messages and sends them to the brain. In the commonest form of AMD—“dry” AMD—the light-sensitive cells in the macula gradually die. In “wet” or “neovascular” AMD (one in 10 cases of AMD, but responsible for 90% of severe AMD-related blindness), abnormal blood vessels grow below the macula. Fluid leaking out of these vessels dislodges and damages the macula, after which loss of vision occurs rapidly. Both forms of AMD destroy the sharp central vision that is needed for reading and driving, leaving only dim, blurred images or a black hole at the center of vision. Neither form can be cured but with wet AMD the loss of vision can sometimes be slowed or halted if caught early by destroying the new blood vessels with laser surgery or a technique called photodynamic therapy or by blocking their formation by injecting special drugs into the eye.

**Why Was This Study Done?:**

No-one knows what causes AMD but factors that increase a person's risk of developing the disease include increasing age, smoking, being white, and a family history of AMD. Recently, researchers have identified several “polymorphisms” (inherited DNA sequence variations that are common within populations) that are associated with AMD. These polymorphisms are in the complement factor H gene (the scientific symbol for this gene is *CFH*) and in a gene region called *LOC387715/HTRA1.* It would be useful to be able to use these risk factors to identify those people at the highest risk of developing neovascular AMD before the disease damages their vision. In this study, the researchers have investigated the association between AMD and polymorphisms in *CFH* and *LOC387715/HTRA1* in more depth. They have then used this new information to build a model of AMD risk that should allow physicians to identify individuals at high risk of developing neovascular AMD.

**What Did the Researchers Do and Find?:**

The researchers catalogued polymorphisms in *CFH* and *LOC387715/HTRA1* in several hundred people with and without neovascular AMD. From these data, they identified three haplotypes (sets of polymorphisms that are inherited as a unit; everyone inherits two copies of each haplotype, one from each parent) in *CFH* that were more common in people with AMD than in those without and two that were associated with a decreased risk of developing AMD. In *LOC387715/HTRA1* they identified one particularly detrimental haplotype. Compared to people without this haplotype, people with one copy of the deleterious haplotype were three times as likely to develop neovascular AMD; people with two copies were thirty times as likely to develop AMD. Smoking history also had a large effect on susceptibility to AMD. The researchers then developed a simple AMD risk scoring system based on *CFH* and *LOC387715/HTRA1*haplotypes and smoking status. From this, they calculated that people with the lowest risk scores have a minimal risk of developing AMD whereas about 15% of people with the highest risk scores are likely to develop AMD.

**What Do These Findings Mean?:**

These findings indicate that it is possible to predict an individual's risk of developing AMD in old age by examining a small number of haplotypes and asking about their smoking status. The model developed by the researchers needs to be validated in other groups of people and may have to be modified if other gene variants that affect the risk of AMD are identified. For now, the results of this research provide physicians with a way to identify those individuals at the highest genetic risk of developing AMD so that they can step up their efforts to persuade these people to avoid smoking. In the future, when effective long-term treatments for AMD become available, the scoring system could also help doctors decide which of their elderly patients should be monitored most intensively for the early signs of AMD so that they can be treated before their vision is irreversibly damaged.

**Additional Information.:**

Please access these Web sites via the online version of this summary at http://dx.doi.org/10.1371/journal.pmed.0040355.

- MedlinePlus provides links to information on macular degeneration and an encyclopedia page on macular degeneration (in English and Spanish)
- Pages on the US National Institutes of Health NIH SeniorHealth site provides text and speech information about AMD
- The US National Eye Institute and the UK Royal National Institute of Blind People also provide information about AMD

## Introduction

Age-related macular degeneration (AMD) is the most common cause of visual disability in the elderly, and is increasing in prevalence in the Western world as the population ages. In addition to age and smoking, genetic factors play a major role in susceptibility to AMD. There is higher concordance in monozygotic than in dizygotic twins [1], and although there is rarely clear Mendelian inheritance, cases tend to cluster in families [2,3]. The search for susceptibility genes has been performed using linkage studies in AMD families, followed by family-based and case-control association studies. Chromosome 1q31 was first implicated in a linkage study involving a large family [4], and the same region was supported consistently in several genome-wide scans based on analysis of smaller multiplex families [5–13]. Several polymorphisms in *CFH,* the gene encoding complement factor H, were found to be strongly associated with AMD, and single nucleotide polymorphism (SNP) rs1061170, encoding a nonsynonymous change from tyrosine to histidine at codon 402, was proposed as the main determinant of disease [14–18]. *CFH* and the closely related genes *CFHR3*, *CFHR1*, *CFHR4*, *CFHR2,* and *CFHR5* are arranged in tandem at the proximal end of a cluster of genes involved in regulation of complement activation. Recently we examined this gene cluster in detail and identified a large deletion that removed both *CFHR3* and *CFHR1* on a strongly protective haplotype [19].The inhibitory activity of complement factor H is controlled by binding of C-reactive protein (CRP), which increases affinity for C3b and leads to down-regulation of complement activity [20]. There are no known coding polymorphisms in the small *CRP* gene, but plasma CRP levels are determined by SNPs within its promoter region [21]. Of most significance in persons of European descent is the triallelic (A/C/T) SNP rs3091244, of which the C allele results in lowest plasma CRP levels and the T allele the highest.

Many previous genome-wide scans also support linkage to Chromosome 10q26, with this region showing the strongest association on meta-analysis [13]. Two reports implicated either *LOC387715* or *PLEKHA1* as a major contributor to AMD susceptibility [22,23], and recently *HTRA1* (which lies 4 kb distal to *LOC387715*) emerged as a leading contender for the second AMD susceptibility gene [24,25]. *HTRA1* encodes a serine protease that is expressed in the retina; however, the mechanism of action of the *LOC387715* gene product remains to be elucidated. The common c.205G>T nonsynonymous coding SNP (rs10490924), which changes alanine to serine at codon 69 of *LOC387715*, is in close linkage disequilibrium (LD) with rs11200638 in the promoter of *HTRA1*, which may regulate its expression [25].

Although earlier studies gave conflicting results, the large Beaver Dam Eye Study in 1993 showed that cigarette smoking increases the risk of exudative (or neovascular) macular degeneration [26]. Other sizeable studies have since confirmed that smoking is a risk factor in all forms of AMD [27–29].

In contrast to most previous studies, which assessed AMD cases ranging from early to late stages of disease, we restricted our cases to those with the blinding neovascular form of AMD in one or both eyes, and compared them to controls with no sign of macular disease. We aimed to refine the association of *CFH* and *LOC387715/HTRA1* with neovascular AMD and to form a model of AMD risk based on inherited variation in these genes in conjunction with smoking status.

## Methods

### Study Population and Diagnostic Criteria

Cases (*n* = 401) with choroidal neovascularisation due to AMD in at least one eye were recruited from ophthalmology clinics in the Royal Victoria Hospital, Belfast, UK, which is the regional referral centre for Northern Ireland. Cases were recruited opportunistically during two intervals, the first between June 2002 and August 2003 (*n* = 305) and the second between June and September 2006 (*n* = 96). Of those invited to participate, 98% agreed. Prior to approaching potential participants, the diagnosis of neovascular AMD was confirmed by clinical examination followed by fluorescein angiography. A control group (*n* = 266) was recruited through random sampling of older adults (aged 65 y and above) from a population-based register, with no exclusion criteria. In this sample the participation rate was ∼50%; those who refused to participate were more likely to be older and female.

Stereoscopic digital fundus photographs were captured at the time of examination, and images were sent to the photographic reading centre for grading using the definitions of the Wisconsin Age Related Maculopathy Grading System. Fundi of controls were free of any drusen, or had fewer than five hard drusen of diameter less than 63 microns. They had no focal pigmentary irregularities (i.e., hyperpigmentation or hypopigmentation). All participants were from Northern Ireland and described themselves as of European descent. Current smoking status and smoking history were obtained through the use of a questionnaire previously validated in the Whitehall study, with participants classified into never smokers, ex-smokers or current smokers [30]. Smoking prevalence data for the UK population in 1974 and 2003 were obtained from the General Household Survey [31], UK Office for National Statistics.

Informed written consent was obtained from all subjects involved (Text S1). Our study was approved by the Research Ethics Committee of the Queen's University of Belfast (Text S2).

### Genotyping

DNA was extracted from peripheral blood leucocytes or frozen buffy coat samples using standard protocols. High-throughput SNP genotyping was outsourced and based on Illumina bead technology (Illumina, San Diego, United States), supplemented in-house with multiplex PCR and primer extension methodology (ABI Snapshot). Rs11200638 was typed by sequencing. All primer sequences are available from AEH. Genotype data were entered in linkage format into Haploview [32] for automated analysis of *CFH* and *LOC387715* haplotypes. Scoring risk at the *CFH*-related cluster was based on haplotypes of SNPs rs6677604, rs3753396, rs419137, and rs2284664. Nonsynonymous coding SNPs rs1061170 (Y402H) and rs800292 (I62V) were also typed. The three SNPs typed within *LOC387715* (rs10490923, rs2736911, and rs10490924) tagged four common haplotypes of a haplotype block that extended across *LOC387715* to the promoter of *HTRA1*. Genotyping of *CRP* markers rs3091244 and rs1205 was restricted to a subgroup of 170 cases and 170 controls, and four *CRP* haplotypes were assessed. All markers within each of the above haplotypes showed complete LD with *D*′ = 1, therefore haplotypes could be assigned with confidence. *LOC387715/HTRA1* haplotypes consisted of rs10490924 and rs11200638 (*D*′ = 0.98; *r* ^2^ = 0.93), and were assigned using PHASE [33].

### Statistical Analysis

The statistical analyses and model building were preplanned before collection of genotype data. Allele frequency differences in cases and controls were assessed by performing Pearson's Chi-square test of association. Each *CFH* haplotype was entered into the logistic regression model as a count (0, 1, or 2) depending on the number of copies of the haplotype an individual carried. To avoid collinearity problems the lowest risk haplotype was omitted from the model so that odds ratios (ORs) were measured relative to homozygote carriers of this haplotype. An exception to this approach was made for analysis of the *LOC387715* and *LOC387715/HTRA1* region where increased risk of AMD was confined to one haplotype, with a much higher risk in homozygote carriers than predicted by the multiplicative model. For our final risk analysis, the number of copies carried of this detrimental haplotype were coded 0, 1, or 2. Tests for deviation from the multiplicative genetic model and for interactions between genetic risk factors and smoking were obtained using likelihood ratio Chi-squared tests. The fitted logistic model produced ORs that were converted to predicted probabilities of AMD for each possible combination of *CFH* and *LOC387715/HTRA1* haplotypes and smoking status. Conversion was performed using an estimated prevalence of severe AMD of 3% and known frequencies for haplotypes and smoking status, and by assuming that *CFH*, *LOC387715/HTRA1*, and smoking were independently distributed in the UK population. These predicted probabilities were used to sort the combinations in order of increasing predicted AMD risk and then to divide the population into approximate tenths of predicted AMD risk. More detailed statistical information is provided in Table S1.

## Results

### Participant Population

Our study involved 401 patients with end-stage neovascular AMD in at least one eye and 266 age-matched controls with healthy fundi. There was a slight preponderance of females in each group, as expected for this age group (Table S2). Mean age at examination was in the mid-seventies for each group (cases 75.6 years; controls 75.8 years), with individuals ranging in age from 54 to 98 y in the case group and from 66 to 100 y in the control group.

### The *CFH* Region Associates with AMD

Our analysis of the haplotype structure of *CFH, CFHR3,* and *CFHR1* has been reported elsewhere [19] and was supported by final HapMap data [34]. There was complete LD within each block, and a strong spine of LD between the three large haplotype blocks covering these genes (multiallelic *D′* = 0.89 between blocks 1 and 2, and 0.91 between blocks 2 and 3). We typed the five common haplotypes of the second block, which encompassed exon 11 to the end of intron 17 of *CFH,* tagged by SNPs rs6677604, rs3753396, rs419137, and rs2284664. Three haplotypes (1:GACG, 2:GAAG, and 3:GGAG) were associated with increased risk and two (4:GAAA and 5:AAAG) were protective of AMD. The most protective (haplotype 5) was associated with deletion of *CFHR3* and *CFHR1* from block 3.

### The *LOC387715*/*HTRA1* Region Associates with AMD

The extremely strong association of *LOC387715* on Chromosome 10 with AMD was confirmed by genotyping SNPs rs10490923, rs2736911, and rs10490924. These SNPs were in complete LD and four haplotypes were identified. Of most importance was rs10490924, with the risk allele (T) being found only on haplotype 2, and present on 48.9% of AMD and 20.5% of control chromosomes. SNP rs11200638 in the promoter of *HTRA1* was typed and found to be in extremely high LD (*D*′ = 0.98; *r* ^2^ = 0.93) with rs10490924 and with *LOC387715* haplotypes, with the risk allele (A; 47.9% of AMD and 19.4% of control chromosomes) closely associated with rs10490924 allele T and *LOC387715* haplotype 2 (Table S2). All four possible haplotypes of *LOC387715/HTRA1* that combined rs10490924 and rs11200638 were found (1:GG, 2:TA, 3:TG, 4:GA), although haplotypes 3 and 4 were exceedingly rare, totalling 1.6% of all chromosomes. These rare haplotypes were distributed similarly through cases and controls and were merged with haplotype 1 for all further analyses. Based on simple counts of alleles or haplotypes, the *LOC387715/HTRA1* haplotype provided marginally better predictive value than either of the constituent SNPs individually (Table S3). The detrimental *LOC387715/HTRA1* haplotype 2 was present on 47.0% of AMD and 18.2% of control chromosomes. Haplotype data for *CFH* and *LOC387715/HTRA1* and smoking status of our cases and controls are shown in Table S4.

### No Association of *CRP* with AMD

The triallelic SNP rs3091244 within the promoter of the *CRP* gene (which affects plasma levels of CRP) was typed, and rs1205 was also assessed to increase haplotype informativeness for this gene. Four haplotypes were identified. The rarest haplotype (4:AC, associated with high CRP plasma level [21]) was more prevalent in cases; however, the overall haplotype distribution in cases and controls failed to reach statistical significance (*p* = 0.06), and using conventional logistic regression the weak association was attenuated by adjustment for carriage of *LOC387715* haplotype 2 and smoking. There was only weak and nonsignificant evidence of interaction between *CFH* haplotype and *CRP* genotype.

### Association with Smoking

As expected, smoking (*p* < 0.001) and previous history of smoking (*p* = 0.05) were associated with disease status, independent of *CFH* and *LOC387715* haplotype. There was no evidence of interaction between smoking and *LOC387715/HTRA1* haplotype (*p* = 0.42), or between smoking and *CFH* haplotype (*p* = 0.07), or between *CFH* and *LOC387715/HTRA1* haplotypes (*p* = 0.42). Smoking history in the controls correlated closely with the data from the UK General Household Survey with 54% and 55% never smokers, respectively; however, this group contained slightly fewer smokers (12% versus 17%) and more ex-smokers than in the general population.

### Logistic Regression Analyses

Logistic regression analysis was performed to examine the effect of the *CFH* and *LOC387715/HTRA1* haplotypes carried and smoking status on AMD. At the *CFH* locus, haplotype 5 conferred the lowest risk, with haplotype 4 also being protective. Haplotype 2 remained the most detrimental, with carriage increasing the risk of AMD more than 4-fold relative to haplotype 5 (Table 1). Two nonsynonymous coding SNPs, rs1061170 (Y402H) and rs800292 (I62V), located within block 1 of *CFH* [19], were also typed. *CFH* block 2 haplotypes 1 and 2 were strongly associated with the high-risk allele C of rs1061170 (402H), with the T allele associated with haplotypes 3, 4, and 5. The low-risk A allele of rs800292 (I62) was associated uniquely with haplotype 4. The detrimental effect of variation at *LOC387715/HTRA1* on AMD phenotype was mediated through haplotype 2. Unlike *CFH*, there was not a multiplicative effect with carriage of two *LOC387715/HTRA1* risk haplotypes. A single copy of haplotype 2 increased the risk of AMD nearly 3-fold relative to the haplotype 1 baseline (Table 2); however, homozygotes for haplotype 2 had their risk greatly elevated by a factor of 33. Past and present smokers showed a graded increase in risk of AMD compared to those who had never smoked (Table 3).

**Table 1:**
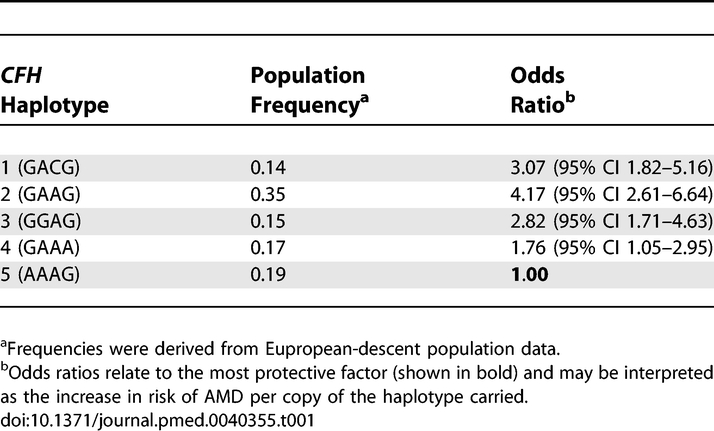
Effect of Haplotype Variation in *CFH* on AMD by Simultaneous Logistic Regression Analysis Including *LOC387715/HTRA1* and Smoking Status

**Table 2:**
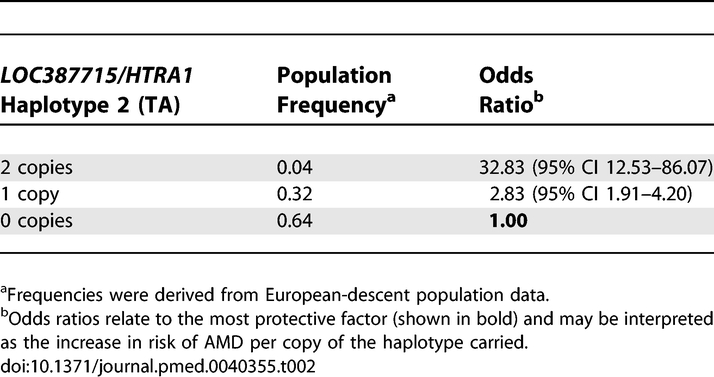
Effect of Haplotype Variation in *LOC387715/HTRA1* on AMD by Simultaneous Logistic Regression Analysis Including *CFH* and Smoking Status

**Table 3:**
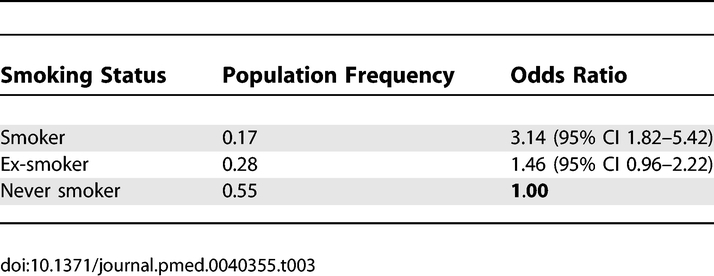
Effect of Smoking Status on AMD by Simultaneous Logistic Regression Analysis Including *CFH* and *LOC387715*/*HTRA1*

### AMD Risk Scoring System

A simple multifactorial AMD risk-scoring system for each individual was developed by multiplying the ORs for genetic effects of each carried haplotype of *CFH* and *LOC387715/HTRA1*, and smoking status.

The overall theoretical distribution of risk for the European-descent population was estimated from haplotype frequencies (derived from reported frequency data available from large studies for the polymorphisms that constituted each haplotype, which did not differ significantly from frequencies found in our control group) combined with UK smoking data since 1974, which classified 55% of our present-aged population as never smokers, 28% as ex-smokers, and 17% as current smokers. This population distribution was split by deciles into ten risk groups for further analysis.

**Figure 1:**
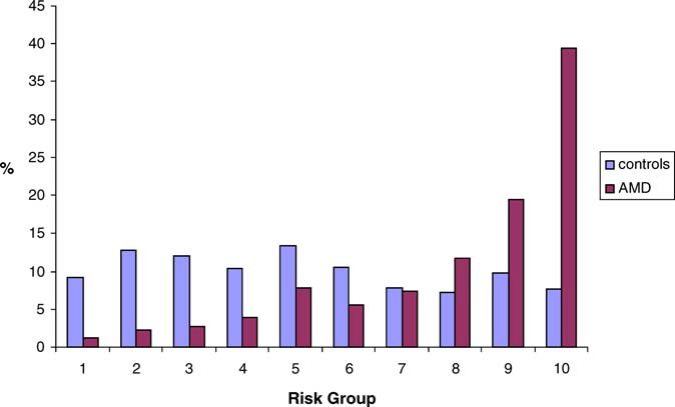
Distribution of AMD Cases and Controls According to Predicted Risk Group Estimated for UK Persons of European Descent The percentages of AMD cases and controls within each risk group are shown.

**Figure 2:**
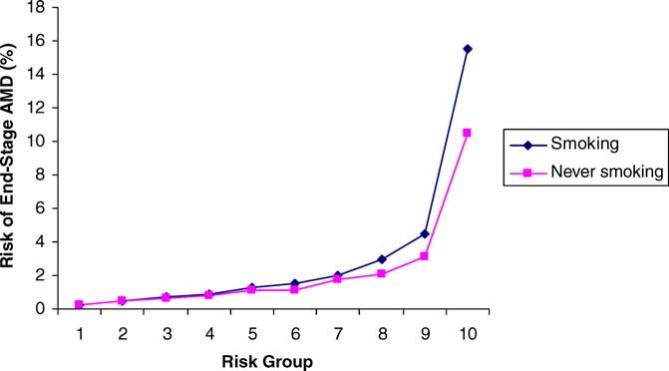
Risk of Developing AMD According to Risk Group in the UK European-Descent Population under the Prevailing Smoking Profile, and if None of the Population Had Ever Smoked

As expected, our controls with no sign of macular disease were biased marginally (and nonsignificantly) in the supernormal direction, with slight clustering in the lower-risk groups, whereas our AMD cases fell predominantly into high-risk groups (Figure 1). The risk of developing end-stage AMD in at least one eye in advancing years can be predicted from an individual's risk score (Figure 2). Those with scores falling within the lowest-risk groups have minimal risk of developing AMD. The overall population prevalence of 3% was exceeded only in the two highest-risk groups, and AMD risk rose steeply to 15.5% for those in the highest tenth of the population.

**Figure 3:**
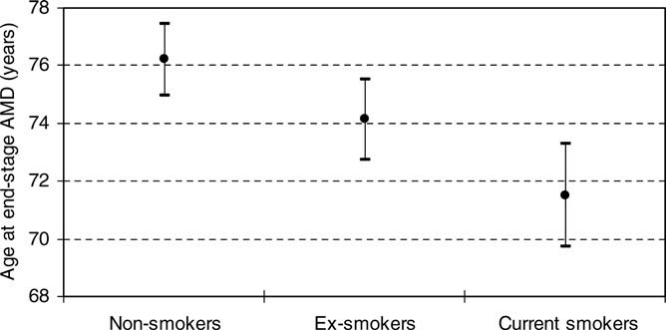
Age of End-Stage AMD According to Smoking Status Shown are means ± 95% confidence intervals of each group.

Overall, genetic factors contributed more than smoking status to risk of AMD, with *LOC387715/HTRA1* proving more influential than *CFH*. The smoking element of the AMD risk score was elevated in our cases compared to controls. Age of onset of neovascular disease in the first eye was known for 296 of the AMD cases. Those who smoked showed earlier onset of end-stage disease than did former or nonsmokers (Figure 3; *F* = 9.81, degrees of freedom = 2,284; *p* ≤ 0.001). There was a similar significant trend to earlier onset with increasing risk group (*F* = 11.97, degrees of freedom = 1,277; *p* ≤ 0.001). When AMD patients were separated into two equal groups based on age of end-stage disease in the first eye, there was an increased effect from *CFH* and smoking in the earlier-onset group and of *LOC387715/HTRA1* in the later-onset group (unpublished data).

## Discussion

Our study assessed the influence of genes in the *CFH* and *LOC387715/HTRA1* regions and smoking status on the etiology of neovascular AMD. We developed a risk model for disease based on comprehensive haplotyping of the genes followed by analysis using logistic regression that also included smoking status. Our model is the first that we know of to take account of haplotypes within the *CFH* and *LOC387715/HTRA1* regions, and allows early identification of those at high risk of developing neovascular AMD in old age. AMD was not inevitable for those in the highest-risk categories, hence exposure to a trigger may be necessary for development of disease.

AMD is one of the most common multifactorial diseases of the elderly. For several years, genes have been thought to play a role in susceptibility. The model generally applied to such complex disorders has been of multiple genes each contributing a small effect to overall risk, in conjunction with environmental factors. This model has been further complicated by the possibility of genes acting with either dominant or negative effects, of gene–gene or gene–environment interactions, and of genetic heterogeneity in which mutations in different genes may have a major influence in different individuals. As is often the case in clinical genetics, a much more straightforward pattern emerges when the possibilities are investigated and some ruled out. Credit must be given to the firm scientific base provided by well-designed linkage studies of families with more than one affected patient from which AMD susceptibility loci have been identified. Although individual studies often identified linkages that have not been confirmed, meta-analyses [13] clearly revealed the regions of Chromosomes 1 and 10 (from which the *CFH* gene cluster and *LOC387715/PLEKHA1/HTRA1* have recently emerged) as major susceptibility loci for all forms of AMD. It is increasingly likely that the genetic component of AMD should be viewed as oligogenic rather than polygenic.

The *CFH* gene cluster comprises five related genes that are all expressed. These genes share a very high level of homology arising from gene duplication, and care must be taken in typing and identifying polymorphic variants at the C-terminal region of CFH and in the *CFH*-related genes. We chose to score risk on the basis of five haplotypes of the most central block, which was in strong LD with the adjacent haplotype blocks in *CFH*, *CFHR3*, and *CFHR1*. Our earlier studies showed strong association between AMD and markers extending across this region [19]. Any coding variants or polymorphisms regulating splicing or expression of these genes may affect risk, and may be based within different haplotype blocks. We also typed Y402H and I62V, but analysis of these common coding polymorphisms did not confer additional information affecting AMD risk. Other studies have limited their analysis to the effect of Y402H, which has been regarded as the main determinant of disease, but this analysis gives an incomplete and inaccurate representation of the region. Only haplotype analysis can assess the effect of combinations of all the coding variants in *CFH* that may contribute to AMD susceptibility. The C allele of rs1061170, which types histidine at codon 402 within the first haplotype block, is associated with a haplotype conferring high risk of AMD, and is coupled with our haplotypes 1 and 2 in the second haplotype block. It is also in complete LD with several other SNPs, one of which has the potential to create a novel splice site and to introduce a new exon containing a premature stop codon. This variant would be expected to result in nonsense-mediated decay of the transcript and reduced CFH levels. Despite being associated with the T allele of rs1061170, haplotype 3 is only marginally less severe in risk than haplotype 1. Haplotypes 4 and 5 are relatively protective. We previously identified a deletion of 85 kb encompassing *CFHR1* and *CFHR3*, which plausibly confers the protective effect of haplotype 5 [19]. The coding variant most strongly associated with haplotype 4 may be the A allele of rs800292, which encodes I62, though this represents a conservative amino acid change. Further work is needed to clarify the molecular bases of the haplotypic effects.

Much interest surrounds the identity of the AMD susceptibility locus on Chromosome 10. Very strong data focus on the protein encoded by the hypothetical gene *LOC387715*, with the alanine-to-serine polymorphism detected by rs10490924 as the most probable causative variant [23]. Alternatives are either *PLEKHA1* or *HTRA1*, which flank the locus; *HTRA1*, which encodes a serine protease, is the more attractive on functional grounds. Another possibility is that the putative exons of *LOC387715* or additional open reading frames in the *HTRA1* promoter region may be included in alternative transcripts of *HTRA1*. The *LOC387715* transcript is exceedingly rare, but has been reported to be present at very low levels in the retina [23]. The markers we typed for this locus identified all common haplotypes of a block that encompassed *LOC387715* to the *HTRA1* promoter. In contrast to the *CFH* gene cluster, the effect of the *LOC387715/HTRA1* region on AMD susceptibility appeared to be mediated through a single detrimental haplotype, with all others being generally protective. In addition to carrying the rare coding variant of rs10490924, the detrimental haplotype was also very strongly associated with several variants in the untranslated region of *LOC387715* and in the promoter of *HTRA1*, and we cannot at present discriminate between the relative merits of variation in *LOC387715* or the *HTRA1* promoter as effectors of AMD susceptibility at this locus.

Further genetic or environmental factors, or their interactions, may be found to affect AMD risk. These factors should be assessed for effect using a regression model that includes all proven genetic and environmental factors, rather than in isolation. Initial reports are prone to inflation to some extent by type 1 error. Conflicting data have been reported about a possible interaction between *LOC387715* and smoking [35,36]. Our evidence does not support an interaction, and we find limited support for an interaction recently reported between *CFH* and *CRP* [37]. Interaction between *CFH* and the genes encoding complement C2 or complement factor B [38], and *CFH* and an excision repair gene named *ERCC6* [39] await confirmation.

Options for reducing the future burden of AMD appear as our knowledge increases. Although smoking status is of less importance than the two main genetic factors, it is a relatively straightforward risk to target in individuals with high genetic load. Our data show that if none of our population had smoked, there would have been an overall reduction of 33% in AMD today. Our current and ex-smokers lost vision at an earlier age, so it is probable that our calculations underestimate the benefit to the population from nonsmoking. We did not assess the importance of smoking on earlier stages of AMD; however, delaying either onset of disease or advancement to the blinding end-stage by 5–10 y would have a major impact on prevalence of blindness. It is now possible to identify those at high genetic risk for whom smoking is particularly unwise. In the past, smokers have been prepared to ignore greatly increased risks of lung cancer and other smoking-related diseases, but attitudes to smoking are changing. Relatives of AMD patients are more likely to carry an excess of *LOC387715/HTRA1* and *CFH*-related genetic risk factors. Those who are shown to be at high risk, with relatives who have lost their vision, may be more prepared to heed advice to refrain from smoking than the general population.

Gene therapy to repair genetic defects is still in the experimental phase and is largely unproven. Of great promise are methods aimed at gene silencing by degradation of RNA using short interfering double-stranded RNA molecules. Deletion of *CFHR1* and *CFHR3* from the *CFH*-related gene cluster is strongly protective against AMD, hence these genes make excellent targets for silencing in future studies aimed at reducing the burden of disease.

### Study Limitations

We assessed the genetic factors and smoking history, which are generally accepted as conferring high population-attributable risk for AMD. The model we developed to estimate risk of neovascular AMD best fits our data, and it is important that it should be validated in independent datasets of similar phenotypes. It is possible that rare variants in other genes may be found to be highly influential in a small proportion of the population, and may cause AMD in a few individuals predicted to be at low risk using our present model. Genes with a modifying effect on AMD risk may also be identified. In either case, our model for prediction of AMD risk may be modified easily. *CFH* may also affect risk of myocardial infarction [40], and we made no attempt to adjust for this condition or for smoking-related diseases that affect longevity.

## Supporting Information

Table S1 Supplementary Statistical Data (34 KB DOC)

Table S2 Combinations of Genotypes at rs10490924 and rs11200638 (25 KB DOC)

Table S3 Comparison of rs11200638, rs10490924, and the Combined *LOC387715/HTRA1* Haplotype of These Markers Analysis is based on allele or haplotype counting and does not account for the nonadditive effect at this locus.
(28 KB DOC)

Table S4 Summary of Gender, Smoking, and Haplotype Data for AMD Cases (*n* = 401) and Controls (*n* = 266) (44 KB DOC)

Text S1 Consent Form (24 KB DOC)

Text S2 Ethical Agreement (276 KB TIFF)

## Abbreviations

AMD: age-related macular degeneration
CFH: complement factor H
CI: confidence interval
CRP: C-reactive protein
LD: linkage disequilibrium
OR: odds ratio
SNP: single nucleotide polymorphism
