# Supplementary material for: Neovascular Age-Related Macular Degeneration Risk Based on CFH, LOC387715/HTRA1, and Smoking
Source: PLoS Med. 2007 Dec 27;4(12):e355. doi: 10.1371/journal.pmed.0040355 (PMC2222948; doi:10.1371/journal.pmed.0040355)
Supplement: Table S1 — (34 KB DOC) [file pmed.0040355.st001.doc]

# **Notes for AMD risk model**

A Smoking (coded as 0=Never, 1=Ex-smoker, 2=Current smoker)

B *LOC387715/HTRA1* two loci, haplotypes coded as 1 (GG, TG, GA) and 2 (TA)

C *CFH* four loci, haplotypes coded as 1=GACG, 2=GAAG, 3=GGAG, 4=GAAA, 5=AAAG

D Frequency of smoking from the UK General Household Survey as in Table 3

E Frequency of *LOC387715/HTRA1* genotype assuming Hardy Weinberg equilibrium and using haplotype frequencies in Table 2

F Frequency of *CFH* genotype assuming Hardy Weinberg equilibrium and using haplotype frequencies in Table 1

G Frequency of the combination assuming independence (=D×E×F) and adding to 100%

H Odds ratio for smoking as in Table 3

I Odds ratio for *LOC387715/HTRA1* multilocus genotype derived from odds ratios in Table 2

J Odds ratio for *CFH* multilocus genotype derived from odds ratios in Table 1

K Odds ratio for the combination predicted by logistic model (=H×I×J)

L Absolute prevalence risk obtained by multiplying odds ratio for the combination (K) by baseline risk of 112.4 per 100,000 population (chosen to give prevalence of 3%)

M Absolute prevalence risk weighted by frequency of the combination (=L×G)

N Excess risk obtained by subtracting from the absolute risk (L) the baseline risk of 112.4 per 100,000 population

O Excess risk weighted by frequency of the combination (=N×G)

P Tenth of predicted risk in population obtained by sorting columns in order of increasing absolute risk (L). Deciles were placed at 11.27; 21.7; 32.15; 40,12; 49.2; 59.1; 70.79; 81.05; 89.94, with later adjustment of data to rebase to 10% in each risk group

**Notes for logistic regression**

The model used may be expressed as

$$\log\frac{\pi^{*}_{(j,k,l,m)}}{1-\pi^{*}_{(j,k,l,m)}}=\beta_{0}+\beta_{jk}^{CFH}+\beta_{l}^{LOC/HTRA1}+\beta_{m}^{Smoking}$$

where $\pi^{*}_{(j,k,l,m)}$ denotes the probability that a subject with *CFH* genotype *jk*, *LOC/HTRA1* genotype *l* and Smoking category *m* is a case

$\beta_{0}$ is a constant representing the baseline risk

$\beta_{jk}^{CFH}$ is the coefficient associated with *CFH* genotype *jk* and is the sum of $\beta_{j}^{CFH}$and $\beta_{k}^{CFH}$, the logarithms of the odds ratios shown in Table 1 *(j,k*=1,2,3,4,5)

$\beta_{l}^{LOC/HTRA1}$ is the coefficient associated with number of copies of *LOC387715/HTRA1* haplotype 2 carried (*l*=0,1,2)

$\beta_{m}^{Smoking}$ is the coefficient associated with smoking category *m* (*m*=0,1,2)

The $\beta$ coefficients are usually expressed as $\exp(\beta)$and interpreted as odds ratios.
