# Supplementary material for: Neovascular Age-Related Macular Degeneration Risk Based on CFH, LOC387715/HTRA1, and Smoking
Source: PLoS Med. 2007 Dec 27;4(12):e355. doi: 10.1371/journal.pmed.0040355 (PMC2222948; doi:10.1371/journal.pmed.0040355)
Supplement: Table S2 — (25 KB DOC) [file pmed.0040355.st002.doc]

|  |  |  | rs11200638 | |
| --- | --- | --- | --- | --- |
|  |  | GG | AG | AA |
|  | GG | 284 | 3 | 0 |
| rs10490924 | GT | 7 | 251 | 1 |
|  | TT | 1 | 9 | 111 |
