# Supplementary material for: Neovascular Age-Related Macular Degeneration Risk Based on CFH, LOC387715/HTRA1, and Smoking
Source: PLoS Med. 2007 Dec 27;4(12):e355. doi: 10.1371/journal.pmed.0040355 (PMC2222948; doi:10.1371/journal.pmed.0040355)
Supplement: Table S3 — Analysis is based on allele or haplotype counting and does not account for the nonadditive effect at this locus. (28 KB DOC) [file pmed.0040355.st003.doc]

|  | **AMD CASES** | **CONTROLS** | **χ^2^** | **p** | **ODDS RATIO** |
| --- | --- | --- | --- | --- | --- |
| **rs11200638** | 418:384 | 429:103 | 112.2 | 3.18x10^-26^ | 3.83 (2.94-4.98) |
| **rs10490924** | 410:392 | 423:109 | 109.9 | 1.02x10^-25^ | 3.71 (2.86-4.81) |
| ***LOC387715/HTRA1* haplotype** | 425:377 | 435:97 | 115.6 | 5.80x10^-27^ | 3.98 (3.04-5.21) |
