# Supplementary material for: Neovascular Age-Related Macular Degeneration Risk Based on CFH, LOC387715/HTRA1, and Smoking
Source: PLoS Med. 2007 Dec 27;4(12):e355. doi: 10.1371/journal.pmed.0040355 (PMC2222948; doi:10.1371/journal.pmed.0040355)
Supplement: Table S4 — (44 KB DOC) [file pmed.0040355.st004.doc]

|  | **AMD CASES** | **CONTROLS** |
| --- | --- | --- |
| **Male** | 163 | 123 |
| **Female** | 238 | 143 |
|  |  |  |
| **Never smokers** | 151 | 124 |
| **Ex-smokers** | 137 | 80 |
| **Current smokers** | 93 | 27 |
| **Missing smoking data** | 20 | 35 |
| **Haplotypes:** |  |  |
| ***CFH* 11** | 9 | 6 |
| ***CFH* 12** | 54 | 20 |
| ***CFH* 13** | 32 | 13 |
| ***CFH* 14** | 18 | 7 |
| ***CFH* 15** | 12 | 15 |
| ***CFH* 22** | 69 | 20 |
| ***CFH* 23** | 81 | 30 |
| ***CFH* 24** | 46 | 35 |
| ***CFH* 25** | 28 | 32 |
| ***CFH* 33** | 12 | 9 |
| ***CFH* 34** | 11 | 21 |
| ***CFH* 35** | 11 | 17 |
| ***CFH* 44** | 7 | 12 |
| ***CFH* 45** | 9 | 20 |
| ***CFH* 55** | 2 | 9 |
|  |  |  |
| ***LOC387715/HTR* 11** | 124 | 171 |
| ***LOC387715/HTR* 12** | 172 | 89 |
| ***LOC387715/HTR* 22** | 105 | 6 |
