# Supplementary material for: Neovascular Age-Related Macular Degeneration Risk Based on CFH, LOC387715/HTRA1, and Smoking
Source: PLoS Med. 2007 Dec 27;4(12):e355. doi: 10.1371/journal.pmed.0040355 (PMC2222948; doi:10.1371/journal.pmed.0040355)
Supplement: Text S1 — (24 KB DOC) [file pmed.0040355.sd001.doc]

CONSENT FORM

ANALYSIS OF GENETIC SUSCEPTIBILITY FACTORS IN AGE RELATED MACULAR DEGENERATION (AMD)

I (name) ………………………………………………………………..

of (address) …………………………………………………………………………..

…………………………………………………………………………..

consent to take part in the above study, the nature and purpose of which have been explained to me. Any questions I wished to ask have been answered to my satisfaction. I understand that refusal to consent would in no way affect the care I receive as a patient.

I understand that I have Age Related Macular Degeneration (AMD) / am a normal control (delete as applicable)

If I am a normal control, I do/do not agree that DNA from my blood sample may be used as a control for other genetic studies. I understand that it will be stored as an anonymous sample with my sex and date of birth.

Signed (Volunteer) …………… Date …………………………..

Date of birth ……………………… Sex …………………………...

(Investigator) ……………………… Date …………………………
